# Sentinel lymph node mapping using indocyanine green in patients with uterine and cervical neoplasms: restrictions of the method

**DOI:** 10.1007/s00404-019-05063-6

**Published:** 2019-02-14

**Authors:** M. Bedyńska, G. Szewczyk, T. Klepacka, K. Sachadel, T. Maciejewski, D. Szukiewicz, A. Fijałkowska

**Affiliations:** 1 Department of Obstetrics and Gynecology, Institute of Mother and Child, Kasprzaka 17a St., 01-211 Warsaw, Poland; 2 Department of General and Experimental Pathology, Medical University of Warsaw, Warsaw, Poland; 3 Department of Pathology, Institute of Mother and Child, Warsaw, Poland; 4 Department of Cardiology, Institute of Mother and Child, Warsaw, Poland

**Keywords:** Endometrial cancer, Cervical cancer, Sentinel lymph node, Indocyanine green

## Abstract

**Purpose:**

To establish the surgical, demographic and histopathological factors associated with inaccurate sentinel lymph nodes (SLNs) identification using indocyanine green (ICG) and near-infrared (NIR) fluorescence imaging in uterine and cervical neoplasms during both open and laparoscopic surgery.

**Methods:**

We reviewed patients with atypical endometrial hyperplasia (AEH), clinical stage I and II cervical cancer or uterine malignancies who underwent primary surgery with SLN mapping between September 2015 and January 2018. An analysis of patients’ demographics, tumor factors and surgical approach was conducted. Bilateral and overall detection rates were calculated and univariate analysis was performed to estimate factors associated with failed SLN mapping.

**Results:**

A total of 32 patients with uterine and cervical neoplasms were included in the study. The overall detection rate of the SLN was 84% and bilateral detection rate was 75%. There were no statistically relevant differences in overall and bilateral SLN detection rates by BMI, surgical approach or age. Regarding endometrial cancer, there were no differences in SLN detection rates when comparing tumor grade, histology nor myometrial invasion. For SLN detection failure, only the presence of metastatic lymph nodes and lack of surgical experience significantly increased the disability to detect SLNs (*p* = 0.03, *p *= 0.04, respectively).

**Conclusions:**

SLN mapping technique using NIR fluorescence imaging with ICG appears to be accurate method in most of the patients with cervical or endometrial carcinoma, regardless of demographic characteristics, tumor-related features and surgical approach. Surgeons’ expertise in that field allows obtaining excellent detection rates.

## Introduction

Surgery plays a fundamental role in the treatment of endometrial and cervical cancer. Since lymph node status is one of the most crucial prognostic factors among these patients, lymphadenectomy remains an important step in surgical management; however, it might be related to several complications such as nerve injury, extensive bleeding, lymphedema or lymphocyst formation [1–4]. Recent studies suggest that despite its position in surgical staging, lymphadenectomy itself does not increase an overall survival among patients [5, 6].

Sentinel lymph node (SLN) concept was initially applied by Cabanas in 1977 in patients with penile carcinoma. Since then, it has been incorporated into management of various cancer and become the standard of care for treatment of breast and melanoma tumors. In gynecologic cancer, it is also widely approved for vulvar cancer and recently has gained rising acceptance in endometrial and cervical carcinomas [7, 8].

Over the past few years, instead of systemic lymph node dissection, SLN biopsy has emerged as one of the most promising methods to achieve tailored surgery among patients with uterine or cervical cancer. Different types of dyes and tracers for SLN mapping were explored through that time. Researchers have reported on their experiences with pelvic lymphatic mapping using radiocolloids [Technetium-99 (Tc-99)] and colorimetric imaging with blue dyes [isosulfan blue (ISB), patent blue, and methylene blue] [9–14].

Recently, fluorescence imaging utilizing light at the near-infrared (NIR) spectrum (700–900 nm) has been described in image-guided oncologic surgery for multiple indications. Indocyanine green (ICG) is the most clinically valuable agent for NIR lymphatic mapping and its usage has been reported in SLN mapping of breast, skin and gastro-intestinal carcinomas with superior safety profile [15]. It also appears to provide a favorable technique for SLN mapping in endometrial and cervical malignancies obtaining excellent detection rates [16]. Since lymphatic drainage in the pelvis is not unilateral, bilateral SLN detection rates are the most clinically relevant. For that reason, credible SLN mapping for cervical and endometrial cancers must reflect bilateral mapping [17].

The aim of this study was to estimate the surgical, demographic and/or tumor-related factors that may affect the ability to accurately identify SLNs using ICG and NIR fluorescence imaging in uterine and cervical neoplasms during both open and laparoscopic surgery.

## Materials and methods

A retrospective analysis of patients with atypical endometrial hyperplasia (AEH), clinical stage I and II cervical cancer or uterine malignancies undergoing SLN mapping at our institution between September 2015 and January 2018 was conducted.

Coexistence of endometrial carcinoma (EC) in patients preoperatively diagnosed with AEH has been shown in several studies [18, 19]. Therefore, patients with AEH were also included in the study.

### SLN mapping technique

In every case, SLN mapping was performed as follows: injection of dye was performed after induction of anesthesia. Intracervical ICG was the utilized fluorophore. The concentration used was 0.5 mg/ml. Four milliliters of this ICG solution was injected into the cervix alone divided into the 3- and 9-o’clock positions, with 1 ml deep into the stroma and 1 ml submucosally on each side [20, 21].

All patients underwent an attempted sentinel lymph node biopsy. Irrespective of the surgical approach (laparotomy or laparoscopy) inspection of the abdominal cavity was the first point of the procedure. SLNs were visualized using ICG camera platform (KARL STORZ GmbH & Co. KG, Tuttlingen, Germany) applicable for laparoscopic and open surgery. Identified SLNs were separately harvested and sent to histological examination. Surgical procedures were carried out upon preoperative histological and clinical assessment as follows.

### Lymph node dissection

Systematic pelvic lymph node dissection (PLND) and paraaortic lymph node dissection (PALND) were performed if the patient with endometrial malignancy had one or more of the following characteristics: preoperative type II endometrial cancers (clear cell or serous), grade 3 endometrioid carcinomas or grossly enlarged lymph nodes in pelvic or paraaortic region suspicious for malignancy on preoperative imaging. Finally, it was conducted in six patients—in other five high-risk patients meeting mentioned criteria PALND was abandoned due to other comorbidities and severe obesity; therefore, PLND was performed solely in that cases.

Systematic PLND alone was performed in five of cervical cancer patients and in five patients with endometrial cancer mentioned above.

In all other patients, lymph node assessment was completed after SLN biopsy.

### Surgical procedures

#### Endometrial malignancies

Patients with a preoperative endometrial biopsy consistent with high grade disease (grade 3 endometrioid, clear cell or serous) or suspicious pelvic lymph nodes on preoperative imaging (CT scan) subsequently underwent total hysterectomy and full PLND and PALND (in some cases). Laparotomy was the approach of choice when PALND was performed despite two cases.

Patients with AEH, grade 1 or 2 endometrioid histology or low-grade endometrial stromal sarcoma (LGESS) underwent SLN dissection followed by hysterectomy. In this group of patients, laparoscopy was the leading access omitting one case when it was abandoned due to anesthetic reasons.

#### Cervical cancer

Patients with preoperative clinical stage I or II cervical cancer underwent C1/C2 type radical hysterectomy according to Q-M classification followed by pelvic lymphadenectomy. Laparotomy was performed in majority of cases: that approach was chosen a priori when tumor was macroscopically visible preoperatively and in case of woman with cancer diagnosed during pregnancy and in that patient hysterectomy was performed perinatally. In one case conversion to laparotomy was indispensable according to anesthetic reasons.

Bilateral salpingoophorectomy was performed in majority of cases [25]. At least one ovary was left in four patients and three patients underwent ovarian transposition. Exact operation types are delineated in Table 1.

**Table 1:** An extent of surgical procedures performed

Total hysterectomy	
+ SLN biopsy alone	15
+ PLND	2
+ PLND + omentectomy	2
+ PLND + PALND	2
+ PLND + PALND + omentectomy	2
+ PLND + PALND + omentectomy + bladder peritonectomy	1
Radical hysterectomy	
+ SLN biopsy alone	1
+ PLND	6
+ PLND + PALND	1

*SLN* sentinel lymph node, *PLND* pelvic lymph node dissection, *PALND* paraaortic lymph node dissection

### Statistical analysis

Statistical analysis was performed using Dell Inc. (2016) and Dell Statistica (data analysis software system), version 13. Univariate analysis with Chi square test was performed to evaluate factors associated with failed SLN mapping (*p* was deemed significant if < 0.05).

## Results

Thirty-two patients with uterine and cervical neoplasms were included in the study and their clinicopathologic characteristics are summarized in Table 2.

**Table 2:** Clinicopathologic characteristics of patients

Patients (*N* = 32)	
Median age, years (range)	61.5 (37–85)
Median BMI (range)	30.5 (19–51)
Histology—postoperative diagnosis	32
Atypical hyperplasia/intraepithelial neoplasia	2
Endometrioid adenocarcinoma of the uterus	14
Grade 1, 2	8
Grade 3	6
Serous/clear cell carcinoma of the uterus	9
Low grade endometrial stromal sarcoma of the uterus	1
Myometrial invasion assessment in uterine carcinoma	
< 50%	13
> 50%	10
Cervical carcinoma	6
Squamous cell carcinoma	4
Adenocarcinoma	2
FIGO final stage	
Preinvasive disease (endometrial intraepithelial neoplasia)	2
Endometrial cancer	
IA	13
IB	8
IIIB	1
IIIC	1
Low-grade endometrial stromal sarcoma	
IA	1
Cervical cancer	
IB1	4
IIA1	2

The median age of patients was 61.5 (range 37–85) and the median body mass index (BMI) was 30.5 kg/m^2^ (range 19–51). The median operative time was 262 min (range 145–460) and the median estimated blood loss was 300 ml (range 100–500 ml).

Cervical ICG injections and SLN biopsy were performed in 22 patients during laparoscopy and other 10 patients during open surgery, including 23 patients with a preoperative diagnosis of uterine malignancies, 3 with AEH and 6 with cervical cancer.

The most common final histology among uterine malignancies was endometrioid adenocarcinoma. Clear cell or serous carcinoma was present in nine postoperative specimens and one patient suffered from LGESS of the uterus.

Four of the six patients with cervical cancer had squamous cell carcinoma with two having adenocarcinoma.

Eleven of the patients with a preoperative diagnosis of endometrial cancer had a grade 1 or 2 lesion, with the remaining having high grade (G3) or non-endometrioid pathologies. Three patients with preoperative grade 1/2 endometrioid carcinoma and the other one with preoperatively recognized AEH turned out to be serous type carcinoma on the final histology and one patient with grade 3 on preoperative samples had clear cell on final histology. One patient with serous type carcinoma from D&C had grade 1 endometrioid type on final examination.

Thirteen of the patients with endometrial cancer had superficial myometrial invasion (less than 50% of myometrial thickness) and the other 10 had deep myometrial invasion (50% or more of myometrial thickness).

The final FIGO stages in endometrial cancers were: IA (12), IB (9), IIIB (1), IIIC (1), LGESS: IA (1) and in cervical cancers, respectively, IB1 (4) and IIA1 (2).

### Lymph node assessment

The median surgical time to complete the SLN mapping was 37 min (range 30–60 min). The overall detection rate of the SLN (uni- or bilateral) was 84% (27/32). The bilateral detection rate was 24/32 (75%) while three cases mapped unilaterally. Any SLN was not identified in five cases. Among patients who successfully mapped the median number of SLNs removed was 2 (range 1–7).

The median pelvic lymph node yield during systemic pelvic lymphadenectomy was 13 and the median paraaortic lymph node count was 7.

Summary of lymph node harvesting is shown in Table 3.

**Table 3:** Lymph node harvesting procedures

Surgical lymph node assessment	32
SLN biopsy only	16
SLN biopsy + PLND	10
SLN biopsy + PLND + PALND	6
Median number of SLNs (range)	2 (1–7)
Median number of PLNs (range)	13 (5–41)
Median number of PALNs (range)	7 (4–20)
Patients with lymph node metastases	2
SLN	0
Non-SLN	2

*SLN* sentinel lymph node, *PLND* pelvic lymph node dissection, *PALND* paraaortic lymph node dissection, *PLN* pelvic lymph node, *PALN* paraaortic lymph node

SLNs were most frequently located in the external iliac region (65.3%), followed by obturator fossa (22.7%), common iliac region (6.7%), presacral region (2.7%), internal iliac region (1.3%) and parametrium (1.3%).

Among all 75 harvested SLNs, none was positive on standard H&E examination.

Failed SLN mapping occurred in five patients: two patients were the two initial cases of SLN mapping performed by our surgeon—one had high grade (G3) endometrioid adenocarcinoma and the other one had serous type carcinoma of the uterus on the final histology. Third patient had adenocarcinoma of the cervix with preoperatively palpable tumor on examination and with bilateral metastatic disease of pelvic lymph nodes on final histology. Fourth case was the patient with high grade (G3) endometrioid adenocarcinoma and FIGO stage IIIC disease with metastatic disease in all four nodal basins (external iliac, obturator fossa, common iliac and paraaortic region) bilaterally. The last patient was obese woman with body mass index 51 kg/m^2^ and with grade 2 endometrial adenocarcinoma on final histology.

Two patients had node positive disease—both failed to map any SLN’s, one was the endometrial cancer patient with positive lymph nodes in all four lymphatic basins and the other one was the patient with adenocarcinoma of the cervix with bilateral pelvic lymph nodes metastatic disease.

There were no false-negative results in women who mapped SLN’s bilaterally or unilaterally.

We evaluate few agents for an association with successful bilateral and overall SLN mapping including patient factors, lymphatic factors, tumor factors, and surgical factors. These data are collected in Table 4.

**Table 4:** SLN detection rate according to alterable and non-alterable factors

Patient characteristics	Patients, *n*	Overall detection, %	Bilateral detection, %	Detection failure, %	Univariate analysis (*p* values)	Bilateral detection failure, %	Univariate analysis (*p* values)
Surgical experience							
≤ 17 SLN procedures performed	17	12/17 (71%)	9/17 (53%)	5/17 (29%)	**0.04**	8/17 (47%)	0.14
> 17 SLN procedures performed	15	15/15 (100%)	15/15 (100%)	0/15 (0%)		0/15 (0%)	
Patient BMI							
≥ 35 kg/m^2^	8	5/8 (62%)	4/8 (50%)	3/8 (38%)	0.11	4/8 50%)	0.16
< 35 kg/m^2^	24	22/24 (92%)	20/24 (83%)	2/24 (8%)		4/24 (17%)	
Tumor histology^a^							
Endometrioid	14	10/14 (71%)	9/14 (64%)	4/14 (29%)	0.11	5/14 (36%)	0.25
Non-endometrioid (LGESS, clear cell, serous)	10	10/10 (100%)	9/10 (90%)	0/10 (0%)		1/10 (10%)	
Tumor grade^b^							
Grade 1, 2	8	6/8 (75%)	6/8 (75%)	2/8 (25%)	0.80	2/8 (25%)	0.51
Grade 3	6	4/6 (67%)	3/6 (50%)	2/6 (33%)		3/6 (50%)	
Myometrial invasion^a^							
< 50%	13	12/13 (92%)	11/13 (85%)	2/13 (15%)	0.75	3/13 (23%)	0.71
> 50%	10	8/10 (80%)	7/10 (70%)	2/10 (20%)		3/10 (30%)	
Surgical approach							
Laparotomy	9	6/9 (67%)	5/9 (56%)	3/9 (33%)	0.16	4/9 (44%)	0.24
Laparoscopy	23	21/23 (91%)	19/23 (83%)	2/23 (9%)		4/23 (18%)	
PLND and/or PALND							
LN positive	2	0/2 (0%)	0/2 (0%)	2/2 (100%)	**0.035**	2/2/(10%)	0.18
LN negative	14	13/14 (93%)	11/14 (79%)	1/14 (7%)		3/14 (21%)	
Age							
> 60	17	15/17 (88%)	12/17 (71%)	2/17 (12%)	0.58	5/17 (29%)	0.63
≤ 60	15	12/15 (80%)	12/15 (80%)	3/15 (20%)		3/15 (20%)	

Statistical analysis was performed with Chi-square test and *p* was deemed statistically significant if less than 0.05 (shown in bold)

*LGESS* low-grade endometrial stromal sarcoma, *PLND* pelvic lymph node dissection, *PALND* paraaortic lymph node dissection, *LN positive* metastases in any lymph node, *LN negative* no lymph node metastases

^a^Tumor histology and myometrial invasion was analyzed in uterine neoplasms only

^b^Tumor grade was assessed for endometrioid type tumors only

There were no statistically relevant differences in overall and bilateral SLN detection rates by BMI (≥ or < 35 kg/m^2^), surgical approach (laparotomy vs laparoscopy) or age (> 60 vs ≤ 60). Regarding endometrial cancer, there were no differences in overall and bilateral SLN detection rates when comparing tumor grade (G1, 2 vs G3), tumor histology (endometrioid vs non-endometrioid) or myometrial invasion (< 50% vs > 50%).

For SLN detection failure, only the presence of metastatic lymph nodes and lack of surgical experience significantly increased the disability to detect SLNs (*p* = 0.03, *p *= 0.04, respectively).

## Discussion

For women with early stage cervical cancer and endometrial cancer, the pathologic status of the lymph nodes is one of the most important prognostic factors and guides postoperative adjuvant therapy [1].

However, the risk of lymph node metastasis in early stage cervical cancer and uterine-confined, low-grade endometrial tumors is relatively low, and the potential morbidity from routine lymphadenectomy (LND) may outweigh population-based clinical benefits.

The influence of routine lymphadenectomy on survival was assessed in two large randomized trials. Benedetti Panici et al. showed higher frequency of metastatic nodes in fully staged patients; however, there were also more cases of lymphedema in those cases. Neither Benedetti Panici nor ASTEC trial group showed overall survival improvement in patients after systematic lymphadenectomy [5, 6].

These findings questioned validity of routine lymphadenectomy in endometrial cancer surgery.

Infrequently, early stage disease is related to nodal metastases. For this reason, over the last decades, there was an increasing need to elaborate a new technique for lymphatic assessment that could substitute inadequate systematic lymphadenectomy and reduce associated morbidity.

Since 2015, SLN procedure has been included in the National Comprehensive Cancer Network guidelines for early stage endometrial and cervical cancer in highly specialized centers, experienced in SLN mapping [22, 23].

SLN mapping may serve as an acceptable compromise in endometrial and cervical cancer surgical staging between no lymph nodes staging and systematic LND. SLN detection rates ranged from 62 to 96% for overall and from 19 to 88% for bilateral detection rate in endometrial cancer and, respectively, from 15 to 100% and from 0 to 93% in cervical cancer according to numerous studies which used various types of dyes and techniques. However, beyond a doubt, this method might be adopted with credibility only in cases with successful bilateral mapping [9, 13].

Barlin et al. reviewed the results of patients who underwent SLN mapping as part of their surgery for endometrial cancer at Memorial Sloan-Kettering Cancer Center. Among 498 women, SLN mapping was performed with blue dye cervical injection. In 81% of cases, at least one SLN was found while bilateral mapping was achieved in 51% of patients. They retrospectively applied the algorithm, which includes the following steps: (1) peritoneal and serosal assessment and washings; (2) retroperitoneal assessment including removal of all detected SLNs as well as all nodes which appear suspicious; (3) If no SLN is detected on one side, side-specific pelvic lymphadenectomy is performed—it is attending’s decision whether or not perform paraaortic LND. The results of application SLN procedure alone compared to the adopting recommended surgical algorithm for all endometrial cancer patients were as follows: SLN accurately recognized 40 from 47 patients with nodal metastatic disease who had at least one SLN found, what gave a 15% false-negative rate. With an implementation of the algorithm, the false-negative rate decreased to 2%, while sensitivity was 98.1% and negative predictive value was 99.8% [24].

Very similar algorithm was applied by Cormier and colleagues in 122 early staged cervical cancer patients. In their SLN biopsy method, intracervical isosulfan blue (ISB) was used in all patients and technetium-99 sulfur colloid (Tc-99) with lymphoscintighraphy was added in some earlier cases. At least one SLN was recognized in 93% of patients, while optimal bilateral mapping was obtained in 75% of cases. There were 21/24 metastatic patients with a positive SLN, resulting in 87.5% sensitivity of SLN procedure. The retrospective algorithm involved removal of any suspicious/enlarged lymph node regardless of mapping, side-specific pelvic lymphadenectomy whether no mapping was present on a hemi-pelvis and parametrectomy performed en bloc with excision of primary tumor. Sensitivity and negative predictive value would reach 100% after applying the algorithm and false-negative rate would be 0 in that particular cohort [14].

Implementing new techniques of surgical staging over the past few years increased overall and bilateral SLN detection rates. However, there is no dispute that a proportion of patients will not achieve successful lymphatic mapping. Why do some patients fail to map?

In our series of 32 patients with AEH, endometrial cancer apparently confined to the uterus and early stage cervical cancer who underwent SLN biopsy we were able to detect at least one SLN in 83% of the patients and bilateral SLNs in 73% of the patients.

We evaluate few factors for an association with successful overall and bilateral SLN mapping including patient factors, lymphatic factors, tumor factors, and surgical factors.

In our study, only the presence of metastatic lymph nodes and lack of surgical experience increased the SLN detection failure which was stated as statistically significant (*p *< 0.05).

### The presence of metastatic disease in the lymph nodes

The relevance of bilateral SLN mapping in cervical and uterine neoplasms results from the fact that the uterus is a midline structure with complex lymphatic anatomy which has already been described and proved with many methods [25, 26]. It was shown in the cervical cancer that the status of the SLN on one side of the pelvis does not reflect the status of the nodes on the contralateral side. The authors suggested that SLN should be detected by each hemipelvis rather than per patient [27, 28].

Plante et al. reported study of 70 early staged cervical cancer patients undergoing laparoscopic radical hysterectomy preceded by SLN mapping performed with isosulfan blue alone or combined approach utilizing Tc-99 and lymphoscintigraphy. Unilateral SLN occurred in 87% of patients while bilateral localization was achieved in 60% of patients. Metastatic lymph nodes were found in 12 patients, eight of them had macroscopically enlarged nodes intraoperatively. In that cohort, SLN was identified only in 56% (9/16 sides) [29]. Similar conclusions were found in Mular’s study. Among 50 cervical cancer patients, metastases were found in 10 patients. In 40% of these patients, no sentinel lymph node was found while overall detection rate achieved in whole cohort was 78% [30].

In our study, systematic pelvic and/or paraaortic lymphadenectomy was performed in 16 of the patients. Metastatic disease in nodal basins was revealed in two cases and both of these cases failed to detect any SLN. We found statistical significance between SLN detection failure among patients with and without metastases in the lymph nodes (*p *= 0.03).

The hypothesis that lymphatic drainage is impeded by cancer cells emboli precluding passing of the injected dye and reaching the node may explain the adversity in detecting enlarged, metastatic nodes. To prove that theory, Leijte and colleagues used bilateral lymphoscintigraphy and SPECT/CT in penile cancer patients with one-sided metastatic palpable groin lymph nodes before contralateral SLN biopsy was done to visualize possible lymphatic obstruction and rerouting of the tracer. In that study, the total lack of lymphatic flow to the palpable, metastatic lymph nodes was seen in 18% of patients while rerouting was observed in 59% of patients [31]. This concept of lymphatic obstruction and rerouting of the lymphatic flow are widely accepted and it was supported by the results of other penile or breast cancer patients’ studies [32, 33]. However, it has been demonstrated that in clinically detectable metastases, it seems credible that this observation can be extrapolated to nonpalpable metastatic lymph nodes. There is still insufficient number of papers referring to that phenomenon in gynecological cancers (endometrial or cervical) nonetheless, algorithms proposed by Barlin in endometrial cancer or Cormier in cervical cancer seem to diminish the possibility of omitting harvesting not detected, metastatic lymph nodes [14, 24].

### Surgical experience

The importance of surgeon’s experience in SLN biopsy has been widely debated in breast cancer and melanoma when accepted number of previous performed procedures before a surgeon can enter patients in clinical trials was 20–30 [34, 35]. In vulvar cancer, it has been recommended a minimum of ten consecutive successful SLN biopsy cases with subsequent inguinofemoral lymphadenectomy and no false negatives before performing SLN procedure solely [8].

Khoury-Collado and colleagues in their prospective study tried to assess how many SLN accomplished cases are needed to achieve high detection rate in patients with endometrial cancer. SLN mapping was performed in 115 endometrial cancer patients by six surgeons while 83% of cases were done by one surgeon. Within first 27 months of the study, SLN was detected in 50 of the 64 operated patients (78%) while during next 15 months overall detection rate significantly increased to 94% (48/51 cases) (*p *= 0.018). One surgeon who performed majority of cases (95 cases) after first 30 patients with SLN identified among 23 (77%) reached 94% overall detection rate in a group of last 65 patients [36].

In previously quoted Plante’s cervical cancer study, there was a significant difference between bilateral detection rate in the first 55 cases (51%) compared with 93% of patients with bilaterally detected SLNs among last 15 cases (*p *< 0.01) [29].

The largest multi-institution prospective study in endometrial cancer SLN biopsy—the FIRES trial—enrolled 385 patients. 18 surgeons from 10 centers in the USA performed SLN biopsy using cervically injected ICG followed by robotic total hysterectomy with pelvic and (in some cases) paraaortic lympadenectomy in 340 of 385 enrolled patients. Successful mapping of any SLN appeared in 293 of 340 cases (86%) and bilaterally mapped SLNs were observed in 52% of cases. This relatively lower than observed in other available studies bilateral detection rate might be explained with the learning curve of the SLN procedure which was the novel technique for majority of surgeons (16/18). Most of the reported series with higher bilateral detection rate were single institution studies with experienced surgeons [37].

The Society of Gynecologic Oncology’s (SGO) and SLN Working Group consensus on SLN mapping and staging in endometrial cancer from April 2017 recommended that due to the absence of precise learning curve in the endometrial cancer surgeons may consider following the American Society of Clinical Oncology (ASCO) guidelines regarding SLN application in breast cancer which suggest completing at least 20 SLN procedures with subsequent lymphadenectomy prior to adopting an SLN algorithm [9].

In our study in the first 17 performed cases, overall and bilateral detection rate were 71% and 53%, respectively. Afterwards, in the following cases bilaterally mapped SLNs were found in every patient. That excellent detection rate in subsequent cases might be elucidated by the learning curve and the apparent number of completed SLN procedures required to achieve competency by the only surgeon in our Centre who used that method. The difference between detection failure in first 17 cases and following 15 cases was found statistically significant (*p *= 0.04).

Additional factors which were also debated by other authors may impact the SLN detection rate; however, in our study we did not identify any findings which might be statistically important.

### Overweight

Tanner et al. assessed a number of hypothesized factors associated with successful mapping in patients with EC or AEH including: patient factors (age, BMI), factors potentially influencing lymphatic drainage (history of appendicitis, endometriosis or prior surgery), tumor factors [tumor size, lymphovascular space invasion, depth of myometrial invasion (%), clinically enlarged lymph nodes, FIGO stage, type I versus II histology], and surgeon factors (use of ISB versus ICG dyes or surgeon experience). On multivariate analysis, only dye choice (ICG over ISB), BMI (< 30 kg/m^2^) and the presence of clinically enlarged lymph nodes maintained a statistically significant association with bilateral SLN mapping rate [38].

The association between failed mapping and BMI is consistent with findings of other studies and is likely a result of impaired visualization caused by increasing adiposity in the lymph node basins.

Jewell et al. in their study assessed SLN’s detection rate in 227 patients with cervical and endometrial malignancies using intracervical ICG alone in all cases and ICG with blue dye (ISB) in 13% cases. Median BMI appeared to impact bilateral mapping—median BMI for unilaterally mapped cases was 34 kg/m^2^ compared to 29.6 kg/m2 for cases which mapped bilaterally (*p *=0.02) [39].

Eriksson and associates analyzed the impact of obesity in SLN mapping in patients with uterine cancer. They reviewed 472 robotic cases undergoing SLN mapping using ICG (66% cases) or blue dye (34% cases). Increased BMI seemed to correlate with unsuccessful mapping (median BMI for bilateral mapping was 29.8 kg/m^2^ while median BMI for patients who did not map was 34.7 kg/m^2^). Higher BMI was connected with a significant decrease in the rate of successful overall and bilateral mapping for both the ICG and blue dye groups. However, in all BMI groups the use of ICG resulted in better bilateral and overall SLN mapping rates compared with the use of blue dye (*p *= 0.002, *p *= 0.011, respectively) [40].

In majority of available studies, ICG was one of the most frequently used dye. NIR fluorescence imaging seems to be capable of a penetration depth of up to 1 cm in tissue. This might be the reason for altered lymphatic tissue visualization due to increased amounts of visceral adipose tissue in patients with obesity—the one of the most important risk factors for uterine cancer [41].

Despite that worsened lymphatic tissue visualization in obese patients ICG appears to be preferred dye in that particular group of patients which was proven by Sinno and associates. They compared fluorometric (ICG) and colorimetric (ISB) SLN mapping during robotic surgery in 71 women with endometrial cancer and AEH. Cases in which ICG was utilized had a higher bilateral and overall SLN detection rate than when ISB was utilized. Further, their outcomes revealed significant adverse association between BMI and SLN mapping only in the ISB group and not in the ICG group (*p *=0.03 vs *p *= 0.14) [20].

As it was mentioned obesity remains one of the most common risk factor for endometrial cancer. Nonetheless, the evaluation of different factors affecting SLN detection rate, as well as overweight, in patients with early cervical cancer was the subject of interest of Salvo et colleagues. The aim of their study was to determine patient- and tumor-related factors which may affect the ability of meticulous SLN identification. The authors performed a retrospective study of 188 patients diagnosed with early stage cervical cancer (stages IA1–IB1 and IIA1) who underwent lymphatic mapping and SLN biopsy. Diverse surgical approach (laparotomy, laparoscopy, or robotic) and various type of dyes (Tc-99, patent blue, ICG, or a combination of tracers) were adopted. At least one SLN was recognized in 90% of cases. None of the analyzed factors like prior conization, tumor size (≥ 2 cm), BMI (> 30 kg/m^2^), and different surgical approaches were associated with aggravated overall SLN detection rate. Bilateral detection rate was 62% and it was worsened only by BMI > 30 kg/m^2^. It should be noted that in this cervical cancer patients study median BMI was 26.6 kg/m^2^ which is generally lower than median BMI in endometrial cancer patients [1].

In our study, a difference between overall detection rate as well as bilateral detection rate in patients with BMI < 35 kg/m^2^ and obese patients BMI ≥ 35 kg/m^2^ was observed; however, it was not statistically significant. The explanation of that outcome may lie in the relatively small number of “extremely” obese patients (or according to WHO class II obesity patients—8 from 32 cases had BMI over 35) and fact that ICG was the only used dye in our study and as it was mentioned it seems to be more adequate dye among patients with overweight.

### Tumor size in cervical cancer

Previously mentioned meta-analysis by Kadkhodayan revealed that large-sized tumors (> 2 cm) patients had lower detection rates than patients with smaller tumors. This might be the result of higher possibility of lymphovascular space invasion (LVSI) which impede the lymphatic flow and tumor size-related injection adversities [13].

AGO trial was the multicenter prospective study of 590 cervical cancer patients. In their study, AGO group allowed for very large tumors in the cohort. In fact, 32% of eligible patients had more advanced disease than stage IB2. 305 included patients had tumor larger than 2 cm in diameter and SLN detection rate in that group equaled 83.6% and was significantly lower compared with patients with smaller tumors (94%) (*p *< 0.001) [42].

Rob et al. performed broad review of literature and concluded that for bigger tumors (> 2 cm) SLN detection rate was 80% while for tumors smaller than 2 cm in diameter it was 95%. It should be noticed that many of the analyzed studies included patients with tumors > 4 cm. As it was mentioned above, failed SLN identification in patients with large tumors might resulted from difficulties in finding adequate injection sites or inability of tracers to reach nodes due to debris or tumor emboli clogging lymphatic channels [43].

In our study, we did not assess a difference in SLN detection rates based on tumor size, since in our centre surgery is performed up to 4 cm tumor diameter only.

### Tumor histology, grading and myometrial invasion in endometrial carcinoma

French prospective trial SENTI-ENDO included 125 FIGO stages I and II endometrial cancer patients who underwent SLN biopsy using dual injection (Tc-99 and patent blue) followed by at least PLND. According to ESMO guidelines, three endometrial cancer risk groups were elicited based on histology: low risk (endometrioid type, stage IA grade 1 or 2), intermediate risk (endometrioid type, stage IA grade 3, or stage IB grade 1 or 2) and high risk (endometrioid type, stage IB grade 3 or non-endometrioid type of any stage and grade). At least one SLN was detected in 111 (89%) patients. No significant differences were observed in the EC risk groups between patients with and without detected SLN (*p* = 0.7). No false-negative cases in patients with endometrioid type EC even in the high-risk group were reported while all three patients with false-negative results (when metastatic disease were found in the lymph-node area without SLN detection) had non-endometrioid EC type with more than 50% myometrial invasion. Based on these findings, the authors suggested that SLN biopsy might not be relevant for this histology; however, the number of patients with non-endometrioid type EC was too low to validate the SLN procedure for this subgroup [44, 45].

Naoura et al. published results of a retrospective multicenter study of 180 early stage endometrial cancer patients who underwent SLN procedure. They were categorized into three risk groups of recurrence based on the same ESMO guidelines. Final histology confirmed low or intermediate risk among 141 patients and high risk among 39 patients. At least, one SLN was detected in 159 of 180 patients (88%) and bilateral SLNs were found in 56% of cases. There were no differences in overall and bilateral detection rates between particular ESMO risk groups: low/intermediate and high-risk groups (respectively, 88%, 88%, 64%, 60*%, p* = ns) [46].

A large meta-analysis prepared by Bodurtha Smith and colleagues allowed 55 eligible studies with total number of 4915 endometrial cancer patients who underwent SLN mapping. Different types of tracers and routes of dye administration were possible in those studies. According to that systematic review, neither non-endometrioid histology nor tumor grade (G3) was significantly associated with worsened SLN detection rates (*p *= 0.515) [11].

Among 23 patients with endometrial carcinoma included in our study, we did not find any relevant differences in overall and bilateral detection rates in endometrioid histology compared with non-endometrioid histology (both *p *= 0.5). Additionally, tumor grade (G1, 2 vs G3) and degree of myometrial invasion (> 50% vs < 50%) were not significantly associated with detection rates (*p* = ns).

### Tracer type and route of administration

Numerous studies compare diverse types of tracers available for SLN mapping (Tc-99, blue dye or ICG) as well as different routes of administration (intracervical or hysteroscopic injection).

Buda et al. verified the impact of use various types of tracers (or their combination) on SLN detection rates among 163 cervical and endometrial cancer patients. Tc-99 radiotracer with blue dye for SLN mapping was used in 77 patients, blue dye only in 38 and ICG alone in 48 patients. The overall detection rate for dual injection was 97%, while for blue dye or ICG alone it was 89% and 100%, respectively. The bilateral detection rate for ICG was 85%—significantly higher than the 58% achieved with dual injection (*p* = 0.003) and the 54% for blue dye alone (*p* = 0.001) [47].

Similar results were obtained by Papadia et al. in their multicenter study which involved 342 women with endometrial cancer. They adopted dual SLN mapping utilizing Tc-99 and blue dye in 147 patients and in 195 cases SLN procedure was accomplished by ICG alone. A statistically relevant difference was registered for the bilateral detection rate in favor of ICG group (73.5% vs 84.1%; *p *= 0.007) [48].

Interestingly, in How’s study each of 100 endometrial cancer patients received a mixture of three tracers (blue dye, ICG and Tc-99) for SLN mapping. At least, one SLN was detected in 92 patients and bilateral detection rate was 76%. There were no relevant differences in overall and bilateral detection rates between ICG and Tc-99 *(p *=0.83, *p *= 0.36, respectively) while ICG achieved significantly higher rates in both overall and bilateral mapping than blue dye (*p *= 0.005, *p *= 0.002, respectively) [49].

Jewell et al. compared SLN detection rates using ICG alone and after adding blue dye in their study which involved 227 uterine and cervical malignancies patients. Since the optimal bilateral mapping of ICG alone was 79% and for ICG and blue dye was 77% which was not statistically significant, the authors concluded that the combined use of ICG and blue dye to improve SLN mapping results appears unnecessary [39].

Meta-analysis comparing ICG and other conventional tracers was made by Ruscito and colleagues. Six studies with overall 538 patients were included. The authors separately analyzed detection rates for ICG vs blue dye, Tc-99 or combination of tracers. Overall and bilateral detection rates were significantly higher for ICG than for blue dye (*p *< 0.0001); however, there were no statistically relevant differences in overall and bilateral detection rates neither for ICG vs Tc-99 nor for ICG vs combination of blue dye and Tc-99 (*p* > 0.2) [50].

Rossi et al. in their prospective study evaluated optimal injection site, either cervical or hysteroscopic, for SLN procedure in endometrial cancer. They compared SLN detection rates accomplished with fluorescence imaging (ICG) during robotic surgery in 29 patients. Overall detection rate after cervical injection (82%) was significantly higher than in the hysteroscopic injection group—33% (*p *= 0.027). The authors suggested that cervical site of injection is more feasible as no additional surgical procedure is required. Further, the anatomic distributions of SLNs after that injection seem to be equivalent to the uterine corpus injections and coherent with the most typical localizations of endometrial cancer lymphatic metastases (external iliac, internal iliac and obturator nodes) [21].

Outcomes of Rossi’s study are supported by Bodurtha Smith meta-analysis. In that review, cervical injection correlated with significantly higher bilateral detection rate (*p *= 0.003) compared with the uterine injection. Although cervical route was associated with a lower rate of SLNs located in paraaortic area, an approximated risk of isolated aortic metastases in preoperative stage I endometrial cancer is estimated at 2–3% [11, 47].

A recent comprehensive review on SLN mapping in cervical cancer, performed by Diab, was composed of 24 studies. According to that paper, SLN mapping with ICG gives higher overall and bilateral detection rates compared with the standard radiocolloid and blue dye technique. Intracervical route has also been established as a precise, safe, and reproducible method in patients with cervical cancer [51].

An acknowledged safety profile is the most crucial benefit of ICG. Serious adverse effects such as anaphylactic reactions have been estimated to occur at 0.05% of patients. Additionally, the application of ICG does not require the injection in a controlled environment and an image acquisition before surgery which importantly reduces the operative time [52].

Since intracervical injection of ICG alone appears to be most attainable method for SLN mapping in endometrial or cervical cancer and is available for minimally invasive approaches, clinicians in recent studies are more prone to use only that tracer. Similarly, in our study that was the only utilized method and considering that fact, we did not compare different types of tracers or injection sites [16].

### Age

The connection between increasing age and declining SLN detection rate was hypothesized to be caused by weakened lymphatic drainage appearing with age. However, only several investigators proposed age as one of the factors that may influence SLN mapping.

139 cervical cancer patients were enrolled in the SENTICOL—French multicenter prospective study. Each patient underwent SLN laparoscopic mapping after patent blue injection and Tc-99. The authors noticed a significantly superior bilateral SLN detection rate among younger patients (*p *= 0.001) [53].

Age was also one of the numerous analyzed features that might be responsible for worsening SLN detection rate in Tanaka’s study where older age (> 60 years old) was associated with significantly decreased detection rate (*p *< 0.01) [54].

Results of our study did not show any relevant differences between the detection rate among older (> 60 years old) and younger patients (*p *= 0.8).

### Surgical approach

Minimally invasive surgical approaches (laparoscopy, robotic-assisted surgery) are becoming more popular not only for benign gynecological diseases but also in oncological field. This might be the explanation of increasing number of SLN mapping cases in gynecological cancer performed via laparoscopy or robotic surgery in current studies.

Although NCCN guidelines did not designate one proper surgical approach for SLN mapping for endometrial and cervical cancer, some authors believe that a clear and wide view by laparoscopy may improve the detection rate [54].

Recently published meta-analysis of 44 studies including 2236 endometrial cancer patients prepared by Lin et al. revealed that laparoscopy and robotic-assisted surgery were associated with higher SLN detection rates when compared with an open surgery [55].

Results of our study are consistent with these findings. Overall and bilateral detection rates were higher in laparoscopy than in open surgery group (91% vs 67%, 83% vs 56%, respectively); however, due to small study size, differences were not statistically important (*p *> 0.05).

## Conclusions

Based on our experience, SLN mapping technique using NIR fluorescence imaging with ICG appears to be an accurate method with high overall and bilateral detection rates in most of the patients with cervical or endometrial carcinoma, regardless of demographic characteristics, tumor-related features and surgical approach. As presence of nodal metastatic disease may impede SLN mapping, an ipsilateral lymphadenectomy should be performed if SLN is not identified on one side. Surgeons’ expertise in SLN mapping field allows obtaining excellent detection rates.
